# Genetic mapping of *Pinus flexilis* major gene (*Cr4*) for resistance to white pine blister rust using transcriptome-based SNP genotyping

**DOI:** 10.1186/s12864-016-3079-2

**Published:** 2016-09-23

**Authors:** Jun-Jun Liu, Anna W. Schoettle, Richard A. Sniezko, Rona N. Sturrock, Arezoo Zamany, Holly Williams, Amanda Ha, Danelle Chan, Bob Danchok, Douglas P. Savin, Angelia Kegley

**Affiliations:** 1 Pacific Forestry Centre, Canadian Forest Service, Natural Resources Canada, 506 West Burnside Road, Victoria, BC V8Z 1 M5 Canada; 2 USDA Forest Service, Rocky Mountain Research Station, 240 West Prospect Road, Fort Collins, CO 80526 USA; 3 USDA Forest Service, Dorena Genetic Resource Center, 34963 Shoreview Road, Cottage Grove, OR 97424 USA

**Keywords:** Disease resistance, Genetic map, Limber pine, Transcriptome *de novo* assembly, White pine blister rust

## Abstract

**Background:**

Linkage of DNA markers with phenotypic traits provides essential information to dissect clustered genes with potential phenotypic contributions in a target genome region. *Pinus flexilis* E. James (limber pine) is a keystone five-needle pine species in mountain-top ecosystems of North America. White pine blister rust (WPBR), caused by a non-native fungal pathogen *Cronartium ribicola* (J.C. Fisch.), has resulted in mortality in this conifer species and is still spreading through the distribution. The objective of this research was to develop *P. flexilis* transcriptome-wide single nucleotide polymorphism (SNP) markers using RNA-seq analysis for genetic mapping of the major gene (*Cr4*) that confers complete resistance to *C. ribicola*.

**Results:**

Needle tissues of one resistant and two susceptible seedling families were subjected to RNA-seq analysis. *In silico* SNP markers were uncovered by mapping the RNA-seq reads back to the *de novo* assembled transcriptomes. A total of 110,573 *in silico* SNPs and 2,870 indels were identified with an average of 3.7 SNPs per Kb. These SNPs were distributed in 17,041 unigenes. Of these polymorphic *P. flexilis* unigenes, 6,584 were highly conserved as compared to the genome sequence of *P. taeda* L (loblolly pine). High-throughput genotyping arrays were designed and were used to search for *Cr4*-linked genic SNPs in megagametophyte populations of four maternal trees by haploid-segregation analysis. A total of 32 SNP markers in 25 genes were localized on the *Cr4* linkage group (LG). Syntenic relationships of this *Cr4*-LG map with the model conifer species *P. taeda* anchored *Cr4* on *Pinus* consensus LG8, indicating that R genes against *C. ribicola* have evolved independently in different five-needle pines. Functional genes close to *Cr4* were annotated and their potential roles in *Cr4*-mediated resistance were further discussed.

**Conclusions:**

We demonstrated a very effective, low-cost strategy for developing a SNP genetic map of a phenotypic trait of interest. SNP discovery through transcriptome comparison was integrated with high-throughput genotyping of a small set of *in silico* SNPs. This strategy may be applied to mapping any trait in non-model plant species that have complex genomes. Whole transcriptome sequencing provides a powerful tool for SNP discovery in conifers and other species with complex genomes, for which sequencing and annotation of complex genomes is still challenging. The genic SNP map for the consensus *Cr4*-LG may help future molecular breeding efforts by enabling both *Cr4* positional characterization and selection of this gene against WPBR.

**Electronic supplementary material:**

The online version of this article (doi:10.1186/s12864-016-3079-2) contains supplementary material, which is available to authorized users.

## Background

*Pinus flexilis* E. James (limber pine) is a keystone five-needle pine species of the subgenus Strobus in mountain-top ecosystems of North America. Its high elevation distribution ranges ~1,600 m to > 3,300 m, much wider than any co-occurring conifer tree species. Populations dominate in dry, rocky, exposed windswept slopes; and its latitudinal range extends from 33°N to 51°N [1]. *P. flexilis* stands show slow growth in diverse environments across the landscape with longevity of some trees surpassing 1500 years, indicating that adaptation to different habitats has equipped this conifer species with a high capacity for physiological plasticity or broad physiological tolerances [2]. The ability of *P. flexilis* to colonize extreme environments and withstand climate variability makes it ecologically important in high elevation ecosystems. However, white pine blister rust (WPBR), caused by a non-native fungal pathogen *Cronartium ribicola* (J.C. Fisch.), threatens the sustainability of this conifer species and other five-needle pines in North America [3]. WPBR, in combination with mountain pine beetle (*Dendroctonus ponderosae*), limber pine dwarf mistletoe (*Arceuthobium cyanocarpum*), and climate change have caused widespread mortality in *P. flexilis* and reduced capacity for forest recovery throughout a significant portion of its range [4–6]; a 40 % loss in basal area of *P. flexilis* is projected by 2030 in the absence of intervention [7].

All native five-needle pines are highly susceptible to WPBR and their wild populations have been impacted to various degrees and at increasing rates [3]. To date, resistance breeding has been the main strategy used for WPBR management. Genetic resistance to WPBR is determined by dominant major resistance (R) genes or multiple genes with minor effects in the five-needle pine hosts [8]. Screening for major gene resistance (MGR) in breeding programs has identified four major R genes against *C. ribicola*, named *Cr1*, *Cr2*, *Cr3*, and *Cr4* in *P. lambertiana* Dougl. (sugar pine) [9], *P. monticola* Dougl. ex D. Don (western white pine) [10], *P. strobiformis* Engelm. (southwestern white pine) [11], and *P. flexilis* [12], respectively. These R genes trigger a hypersensitive reaction (HR)-like defense that usually limits infection to the needles, precluding stem infection. Infection by different virulent inocula indicated that *Cr1*, *Cr2*, and *Cr3* target at different avirulent (*avcr*) alleles [11]. *Cr4* shows resistance to the virulent (*vcr2*) inoculum that overcomes *Cr2* [12]. Although the classic gene-for-gene interaction [13] has been demonstrated in these WPBR pathosystems; neither R genes nor their corresponding *avcr* genes have been molecularly characterized.

Genetic maps of *Cr1* and *Cr2* were developed previously [14–16], but we still do not know whether five-needle pine R genes are different alleles of the same R gene, or different R genes. The majority of characterized plant R genes belong to the super families encoding proteins with nucleotide binding site/leucine-rich repeat (NBS-LRR) or encoding receptor-like kinases (RLKs), which confer hosts complete resistance against various pathogens/pests, including biotrophic fungal pathogens [17]. For development of R gene-targeted DNA markers, NBS-LRR genes have been isolated from *P. monticola* [18] and *P. lambertiana* [19]. DNA markers (including single nucleotide polymorphism-SNPs) of the NBS-LRR genes were used as functional candidates for R gene characterization in the WPBR pathosystem [20]. In the two past decades, various types of DNA markers have been developed and used for genetic studies of WPBR resistance or for construction of genetic maps of five-needle pines [16, 21–25]. However, DNA markers have not been developed in *P. flexilis*, and genetic diversity and molecular mechanisms underlying *P. flexilis* resistance to WPBR remain poorly understood.

Like other *Pinus* species, *P. flexilis* has a huge genome with 1 C of 31.2 pg [26]; the estimate of total genome length is about 30.5Gbp. Although the *P. taeda* draft genome has been assembled [27] and the cost of next generation sequencing (NGS) has decreased, sequencing the *P. flexilis* genome is still a challenging task due to its huge size. Identification of genetic determinants is critical for the development of disease resistant *P. flexilis*. Compared to agricultural plants, conifer breeding is a slow process, because identifying and pyramiding disease tolerance traits is far more challenging in these species with much longer life cycle, and larger but less well characterized genomes. DNA markers closely linked to R genes have the potential to strengthen the efficiency of breeding [28, 29]. In *P. flexilis* and other five-needle pines, resistant parents can be identified relatively quickly if a marker-assisted selection (MAS) tool is available.

This research was undertaken to identify a large number of SNPs in the *P. flexilis* transcriptome *de novo* assembled by RNA-seq analysis. SNPs were then applied to develop high-throughput genotyping arrays for genetic mapping of a *Cr4* linkage group (LG) using populations selected from current breeding and conservation programs in North America. *Cr4* was anchored on a *Pinus* consensus genetic map by comparison with *P. taeda* genetic maps.

## Results

### Phenotypic analysis of genetic resistance to *C. ribicola*

**Fig. 1:**
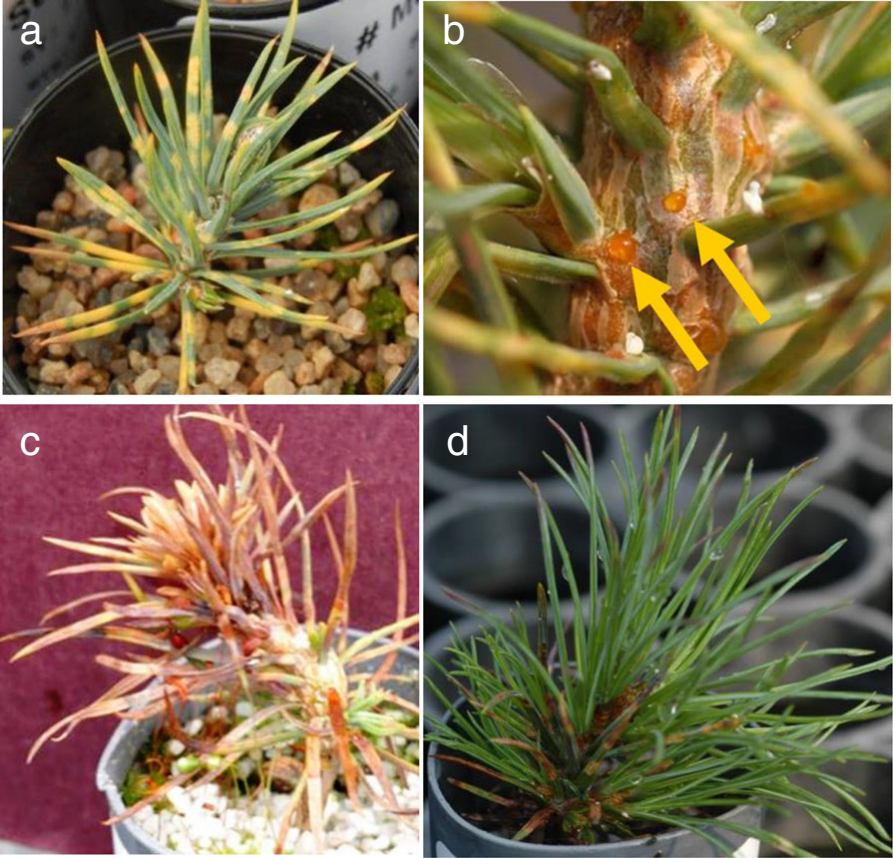
Disease phenology on *Pinus flexilis* seedlings with rust inoculation at 5-months old. **a** Needle lesions (*spots*) at 3 months post-inoculation (MPI); **b** Spermatia (pycnia) evident at 10 MPI, with arrows indicating pycnian drops; **c** Susceptible seedling killed by rust; **d** Resistant seedling growing in the 2^nd^ year

Four seedling families were assessed for phenotypic segregation of *Cr4*-mediated resistance for *Cr4* genetic mapping. Their seed germination rates varied from 38-75 %, and seedling family GE-213, GE-214, LJ-112, and PHA-106 yielded 146, 145, 122, and 156 seedlings, respectively. The inoculation spore density delivered to the seedlings was measured at 11,670 spores/cm^2^ and *C. ribicola* basidiospores showed a germination rate of 97 %. Each seedling was phenotyped via inspections for signs and symptoms of disease and resistance; early inspections revealed *C. ribicola* needle infection spots (lesions) on all seedlings and 99 % of them presented with more than 10 needle infection spots, indicating a successful inoculation that challenged every seedling (Fig. 1). Cankered seedlings with production of spermatia were considered as a confirmed susceptible genotype, its occurrence ranging from 35.9 % (seedling family GE-213) to 61.8 % (seedling family PHA-106) with an average of 52.9 %. Phenotypic segregation for stem-cankered and stem-canker-free was consistent with previous tests [12].

### *P. flexilis* needle transcriptome

RNA-seq analysis on needle tissues generated approximately 68, 72, and 70 million 100 bp PE reads for seedling families MRO-3501, UT-3359A, and NR-3647, respectively. Quality filtering by the read trim procedure removed about 0.3 % of total reads due to low quality. Our *de novo* assembled needle transcriptome using Trinity contained 163,075 transcripts with N50 at 1,861-bp and total transcriptome length of 158.5 Mbp (Additional file 1: Table S1). The mRNA sequences were predicted as expressed from 98,996 unigene sequences. A BLASTx search using the *P. taeda* proteome (84,522 proteins) revealed the transcriptome assembly contained 10,886 unique gene sequence highly conserved between *P. flexilis* and *P. taeda* (E value ≤ 10e-100). A total of 37,294 unique gene sequences showed significantly homology hits (BLASTx E value ≤ 10e-6) to the *P. taeda* proteome. These 37,294 *Pinus* conserved unigene sequences covered a total *P. flexilis* exome sequence length of 42.9 Mbp, which accounts for 0.14 % of the 30.5 Gbp genome of *P. flexilis*. A tBLASTn search of the *P. taeda* protein database (including 84,552 putative proteins) against our *P. flexilis* transcripts revealed that 94.3 % of them had significant homology hits (E < 10e-6) (Additional file 1: Table S2), suggesting that our *de novo* assembled *P. flexilis* transcriptome may have a relatively high coverage.

*P. flexilis* NBS-LRR genes and defense-related genes in response to *C. ribicola* infection were predicted using a BLAST search against the corresponding sequence sets from *P. monticola*. BLASTx analysis (E value ≤ 10e-10) identified 2,654 transcripts encoding NBS-LRR proteins expressed from 792 unigenes, and BLASTn analysis (E value ≤ 10e-100) identified 2,219 transcripts encoding putative WPBR-responsive proteins expressed from 827 unigenes in the *P. flexilis* needles (Additional file 1: Table S2).

**Fig. 2:**
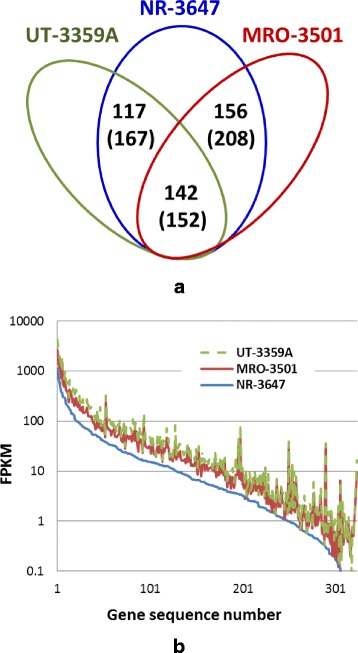
Transcriptome analysis of gene expression levels using RNA-seq. **a** Venn diagrams of differentially expressed genes (DEGs) as detected by comparisons of transcriptomes between resistant (NR-3647) and susceptible (UT-3359A and MRO-3501) seedling families. Numbers of transcripts expressed from DEGs are shown in the parentheses. **b** Expression values (FPKM) of the genes with SNPs selected for design of genotyping arrays. FPKM values were mapped to the genes in order from the largest to the smallest values, using the transcriptome of the resistant seedling family NR-3647 as a reference

Transcriptome comparison analysis identified 415 differentially expressed genes (DEGs) between resistant seedling family (NR-3647) and susceptible seedling families (MRO-3501 and UT-3359A). A total of 527 transcripts were expressed from these DEGs, and 142 DEGs for 152 transcripts were commonly detected in both comparisons of transcriptomes: NR-3647 vs. MRO-3501, or NR-3647 vs. UT-3359A (Fig. 2a). As revealed by GO annotation analysis on DEGs, the three top subcategories under the biological process category were oxidation-reduction process (score 64.3), cellular protein metabolic process (score 53.7), and response to stress (score 43.7). Fifty-three and 25 DEG sequences encoded for putative R-homologs (NBS-LRR and RLK) and pathogenesis-related proteins, respectively. This evidence suggests that DEGs may be involved in disease resistance and/or adaptation to local habitats.

### *In silico* SNP calling

Using our *de novo* assembled transcriptome as a reference, a total of 110,573 SNPs and 2,870 indels were called in the three seedling families, and these polymorphisms were distributed among 21,561 transcripts expressed from 17,041 unique genes. On average, we detected 6.5 SNPs per unigene and 3.7 SNPs per Kb. Of 10,886 *P. flexilis* unigene sequences highly conserved between *P. flexilis* and *P. taeda* (BLASTx E value ≤ 10e-100), 6,584 (~60 % of total) were polymorphic with SNP distribution. A BLASTn search against *P. taeda* mapped gene sequences [30], yielded 2,165 *P. flexilis* polymorphic genes with identical hits (E values < e-100), providing anchored conserved genes for comparative mapping between *P. taeda* and *P. flexilis*.

**Fig. 3:**
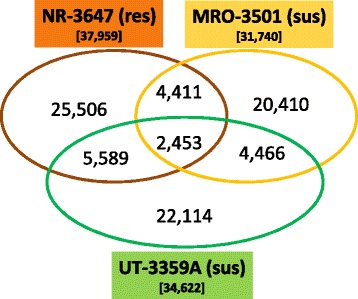
Number of single nucleotide polymorphisms detected in three seedling families of *Pinus flexilis*. The intersecting portions of the Venn diagram illustrate the number of shared loci in pair-wise comparison of seedling families

Nucleotide variation counts detected 31,740, 34,622, 37,959 SNPs; as well as 234, 273, and 515 indels in the seedling families MRO-3501, UT-3359A, NR-3647, respectively. The majority of nucleotide variations (63.9 % to 67.2 % of total SNPs and 76.9 % to 85.0 % of total indels) were seed family-specific. Nucleotide variations shared among seed families were limited. Pair-wise comparison among the three seedling families revealed that only about 20 % of total SNPs from one seedling family were shared with another family (Fig. 3); i.e. most nucleotide variations were localized within individual seedling families. Those MGR seedling family-specific SNPs were potential sites for searching for *Cr4*-linked DNA markers at a next step.

### *Cr4* genetic mapping by high-throughput SNP genotyping

**Fig. 4:**
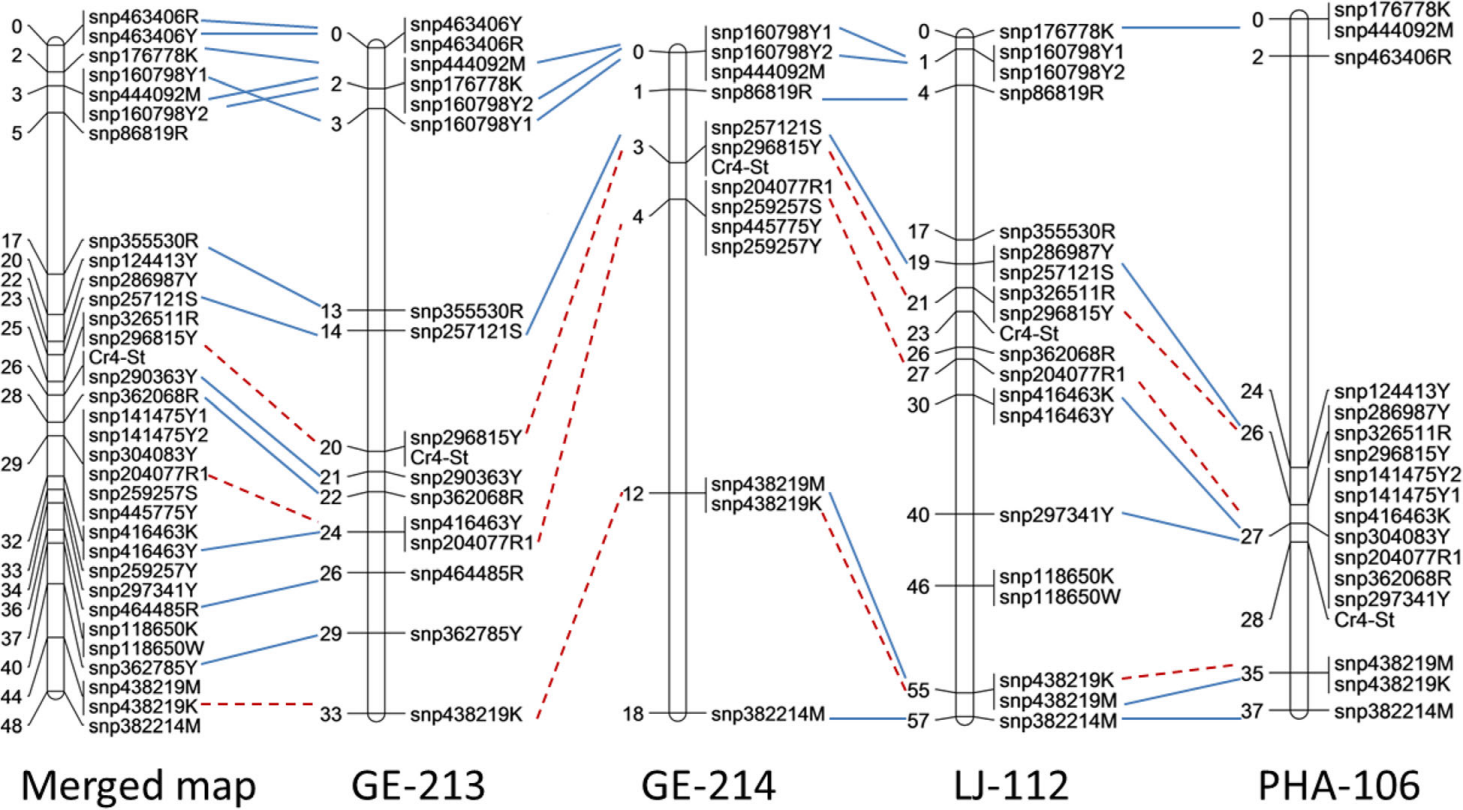
Genetic linkage map of SNP markers in seedling families GE-213, GE-214, LJ-112, and PHA-106. Three SNP markers that segregated in all four genotyped seedling families are shown by *red lines*

A total of 324 *in silico* SNPs were selected in candidate gene groups (NBS-LRR or RLK gene families and putative WPBR-responsive genes) with high conservation among *Pinus*, and used to design Sequenom genotyping arrays. In addition to SNP coverages, gene expression was also considered in SNP selection. As shown in Fig. 2b, a majority of genes with selected SNPs (>83 %) shows good transcript expression (FPKM >1 at least in one seedling family), 21 genes were expressed differentially between resistant and susceptible seed families (*p* < 0.05 with FDR correction). Others were included due to their high homologies with candidate groups. These SNP markers were screened in the seedlings with identified phenotypes following *C. ribicola* infection. Seventy-two SNPs were selected to genotype all megagametophyte samples from four maternal trees. After manually checking SNPs, we constructed individual linkage maps for each maternal tree separately (Fig. 4). Linkage of SNP markers to *Cr4*-mediated phenotypes was significant in all four maternal trees (logarithm of odds-LOD ≤ 6 for GE-213, LOD ≤ 9 for GE-214, and LOD ≤ 10 for LJ-112 and PHA-106). The SNP markers shared among the four maternal trees were aligned manually and a total of 32 SNP markers were found to reside in 25 unique genes (Additional file 1: Table S3).

Among the 32 *Cr4*-linked SNP markers, only three (snp296815Y, snp204077R1, and snp438219K) were shared across all four maternal trees. The same allele at each of these three shared SNP loci was linked in coupling to the resistant allele at the *Cr4* locus in all four families used in the mapping experiments described here. Nine SNP loci (snp160798Y1, anp160798Y2, snp297341Y, snp355530R, snp382214M, snp416463K, snp444092M, snp463406Y, and snp86819R) were mapped in repulsion on the *Cr4*-LGs in different maternal trees. These results indicated high genetic divergence among the maternal trees from different geographical regions.

The order of the SNP markers was highly consistent across the maternal trees. Multiple SNPs from the same gene sequence (for example, snp463406Y and snp463406R) were usually mapped at the same position on the LG. A consensus *Cr4*-LG was constructed by merging four individual maternal maps with a total length of 47 cM and an average density of 1.5 cM per SNP marker. The *Cr4* region was highly saturated; and SNP markers snp290363Y and snp296815Y were closest to *Cr4* with genetic distances at 0.35 cM and 1.00 cM respectively (Fig. 4).

### Comparative genetic map of *Cr4* linkage group

**Fig. 5:**
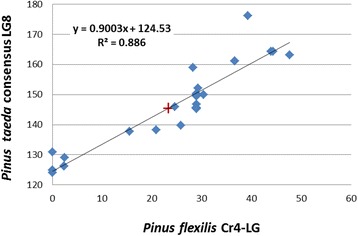
Scatter plot analysis to evaluate the correlation of marker positions of *Pinus* highly conserved genes between *Pinus flexilis* and *P. taeda. P. taeda* gene positions on *Pinus* consensus linkage group 8 were reported previously by Westbrook et al. [30]. The symbol "+" indicates the predicted position of the Cr4 locus

We compared the *Cr4*-LG to *P. taeda* consensus maps reported recently [30]. All 25 genes mapped on the *Cr4*-LG showed identical hits to *P. taeda* genes, with BLASTn e-values ranging from 7.66E-109 to 0 (Additional file 1: Table S4). All 25 *Pinus* highly conserved genes were localized on the *P. taeda* consensus LG8; and 21 of them were mapped with relative positions on LG8. The *Cr4*-LG shows high collinearity when the SNP markers are compared according to the corresponding functional genes they tag (Additional file 2: Figure S1). The relative order of the mapped genes is highly correlated among two *Pinus* species with Pearson correlation *R*^2^ = 0.886 (*p* < 0.00001) (Fig. 5). The comparative mapping anchored *Cr4* on the *Pinus* consensus LG8 at a position around 146.74 cM.

### Functional genes close to *Cr4*

The 25 functional genes localized on the *Cr4*-LG map were annotated, and 21 of them encode proteins with putative biological functions (Additional file 1: Table S4). Several genes close to *Cr4* were revealed with potential roles in genetic resistance against pathogens/pests, and they encoded putative proteins with significant homologies to class IV chitinase, NOTUM homolog isoform x1, zinc finger CCCH domain-containing (C3H-type ZF) protein, benzyl alcohol o-benzoyl transferase (EC: 2.3.1.196), aleurain-like thiol protease (EC: 3.4.22.16), nodulation-signaling pathway 1 (NSP1), and galactinol synthase 1 (GOLS1, EC: 2.4.1.123) (Additional file 1: Table S4). Other annotated genes that were mapped on the *Cr4*-LG included those encoding proteins with homologies to NBS-LRR protein, expansin, SHISA-5, endoglucanase, and protein sensitive to proton rhizotoxicity, suggesting their functions in plant defense responses to environmental stresses.

## Discussion

### Transcriptome SNP development by RNA-seq analysis

In the present study, *Cr4* was mapped using a research strategy that integrated SNP development using RNA-seq analysis and subsequent high-throughput genotyping with only a few hundred *in silico* genic SNP markers. Recently we used this strategy for genetic identification of functional genes in significant linkage disequilibrium with *P. monticola Cr2* for major gene resistance to *C. ribicola* [20]. As the cost is low and affordable for most research labs, this strategy may be useful for similar research on non-model conifer species. The *P. flexilis de novo* assembled transcriptomes and large number of genic SNPs identified in this study may provide useful genomic resources for further investigation of resistance mechanisms and selective adaptation to biotic/abiotic stresses and climate change in other native North American five-needle pines.

Molecular breeding of five-needle pines for resistance to WPBR would benefit from recent advance in conifer genomics studies, including the *P. taeda* genome sequence, the recent release of the *P. lambertiana* draft genome assembly [27] (also see www.pinegenome.org/pinerefseq/), and *de novo* assembled transcriptomes and related NGS data from other five-needle pines [25, 31, 32]. Genome annotation for protein-coding genes in *Pinus* species [27, 33] provides a valuable source of genic DNA markers for comparative analysis among related conifer species, with a high potential to determine candidates for map-based gene cloning and identification of functional markers for marker assisted selection in the WPBR pathosystems. Research approaches for this study have integrated genomic information previously reported in the genus *Pinus* to generate a consensus genetic map for *Cr4*-LG, an important step towards *P. flexilis* molecular breeding and further genomics research on resistance mechanisms.

### Construction of *Cr4*-LG consensus map using functional SNP markers

Traditional DNA markers (such as RAPD, AFLP, and SSR) are developed on random genomic sequences without knowledge of the causal genes. Marker discovery has been greatly improved as NGS technologies have advanced and whole genome or transcriptome sequences in non-model plants have become available. The majority of SNP markers discovered through a strategy of genotyping by sequencing were localized in the non-genic regions [25]. Recently RNA-seq-based transcriptome profiling has been widely used for SNP discovery in forest conifer species [20, 32, 34–37]. SNP markers derived from transcriptomes represent alleles of functional genes with transcript expression. The functional SNPs targeted at exomes may cause amino acid changes in the encoded proteins, with differentiation of protein structures and biochemical properties. This type of DNA marker thus has a potential to provide direct insight into the biological contribution of alleles underlying physiological traits of interest [38].

We constructed a consensus map by genetically localizing *Cr4* using 32 SNP markers that represented 25 unique genes. These *Cr4*-linked genic SNP markers were selected from *in silico* SNPs by transcriptome comparison between *Cr4*-resistant and *cr4*-susceptible seedling families. Common SNP loci among four maternal trees that originated in three different geographical regions were used to build a consensus map for *Cr4*-LG. Comparison of the *Cr4*-LG maps among the four maternal trees revealed highly consistent marker orders for the 25 mapped genes, by which the consensus *Cr4*-LG map was constructed. We observed small discrepancies for the *Cr4* position in four individual *Cr4*-maps, which may not suggest real chromosome rearrangements because different numbers of susceptible samples were calculated in each mapping population. As more recombination events are expected in the larger population resulting from the merging of four maternal trees, the *Cr4* position on the consensus map is theoretically more reliable.

### *Cr4* is anchored on the *Pinus* consensus LG8

Localization of 25 *Pinus* conserved genes in the consensus *Cr4*-LG map gives us a solid framework for further comparative analysis of *P. flexilis* resistance with other pine species. By comparison with recently updated *P. taeda* consensus genetic maps [30], we anchored *Cr4* to the *Pinus* consensus LG8 with a predicted location at 146.74 cM. The highly syntenous relationship between *Cr4*-LG and the *Pinus* consensus LG8 (*R*^2^ = 0.886) will guide fine mapping using larger populations to increase marker density in the genomic region of *Cr4* in a future study. Application of consensus maps with syntenic markers localized *P. lambertiana Cr1* on the *Pinus* consensus LG2 [16], the same LG where *P. taeda Fr1* for resistance to fusiform rust pathogen *Cronartium quercuum sp*. fusiforme (*Cqf*) was localized [27, 39]. *P. monticola Cr2* was localized on *Pinus* consensus LG1 (Liu et al. unpublished data).

Localization of *Cr1*, *Cr2*, and *Cr4* on different LGs (or chromosomes) indicates that R genes against *C. ribicola* have evolved independently in five-needle pines. *C. ribicola* invaded into North America only about one hundred years ago, suggesting that occurrence of the five-needle pine R genes is not caused by pathogen selection pressure and that they may have already been present in wild populations at rare frequencies in North America before the arrival of *C. ribicola*. Furthermore, the maternal trees sampled in this study, except PHA106, are from sites not yet invaded by *C. ribicola*. A further genomic comparison of *Pinus* R genes against *C. ribicola* and *C. quercuum f. sp. fusiforme* would generate new insights into the evolution of the innate immune response within the genus [27].

### *Cr4* functional candidates

Localization of functional genes as well as their SNPs on the *Cr4*-LG facilitates *Cr4* characterization at the molecular level. Genetic mapping of the R gene families is an effective strategy for identification of positional R-gene candidates, development of high-density R-gene genetic maps, and design of diagnostic markers for breeding selection of resistance genotypes and QTL [40]. Several genes with tight linkage to *Cr4* were identified as functional R candidate genes and defence-related genes in the *P. flexilis* immune system.

Contig_463406, encoding a NBS-LRR protein as a homolog of the tobacco N protein against tobacco mosaic virus (TMV), was mapped at a position of ~25 cM from *Cr4*, which indicates that it is not the *Cr4* candidate. However, >60 % of NBS-LRR genes are organized in clusters in angiosperms, and R-genes families have been expanded by lineage-specific tandem duplications with high sequence similarity between duplicated gene copies [41]. Therefore, searching LP-463406 homologs is probably useful in finding other NBS-LRR genes as *Cr4* positional candidates. *P. flexilis* contigs LP-296815, LP-290363, and LP-362068 were localized within about one cM from *Cr4*, providing landmark genes for searching for *Cr4* candidates on the *Pinus* consensus maps. One cM genetic distance probably covers about 10 Mbp of genomic DNA sequence on the *Pinus* physical map [30]. As *P. taeda* and *P. lambertiana* genome draft assemblies are updated with improved completeness, this length of DNA sequence can be scrutinized for members of R gene families (such as NBS-LRR and RLK), which may include the targeted *Cr4* gene itself.

Six functional genes were uncovered in a range of 3 cM from *Cr4* encoding proteins with putative physiological roles in disease resistance, including class IV chitinase, NOTUM homolog, C3H-type ZF protein, benzyl alcohol o-benzoyltransferase, aleurain-like thiol protease, and NSP1. Class IV endochitinaes are a group of pathogenesis-related (PR) proteins functioning in plant defence against pathogens [42]. As glycosyl hydrolases, they catalyze the degradation of chitin, a β-1,4-linked polymer of N-acetylglucosamine (GlcNAc), a major structural component of fungal cell walls. *P. monticola* class IV endochitinases were up-regulated in both resistant and susceptible seedlings post-*C. ribicola* infection [31], and an association study demonstrated their significant contribution to partial resistance to *C. ribicola* [24].

C3H-type ZF proteins may have regulatory functions in mRNA processing during a series of plant developmental and adaptive processes, including plant defense responses to (a)biotic stresses [43]. Most C3H-type ZF genes are regulated by biotic or abiotic stresses in Arabidopsis and rice, suggesting that they may be involved in plant tolerance to stresses [44]. In the *P. monticola* C3H-type ZF family, expression of two members are upregulated in response to *C. ribicola* infection, one responsive in both resistant and susceptible genotypes and another responsive only in *Cr2*-resistant seedlings [20]. A loss-of-function mutant of an Arabidopsis C3H-type ZF protein showed an increased local susceptibility to a fungal pathogen and sensitivity to seed germination in the presence of ABA [45]. Overexpression of a cotton C3H-type ZF gene (*GhZFP1*) in transgenic tobacco increased resistance to a pathogenic fungus (*Rhizoctonia solani*) and enhanced tolerance to salt stress [43].

NOTUM-homologs encode pectinacetylesterase (PAE) in plants and fungi, catalyzing the deacetylation of pectin, a major compound of primary cell walls. Decreased pectin acetylation resulted in increased *Arabidopsis* resistance to microbial pathogens [46–48], suggesting that pectin acetylation may play an important role in plant resistance to pathogens. Transgenic Arabidopsis plants with over-expression of an *Aspergillus nidulans* PAE gene (*AnRAE*) showed reduction of pectin and xyloglucan acetylation and enhanced resistance to *Botrytis cinerea*, which probably is achieved by H_2_O_2_ accumulation, up-regulation of defense-related genes, and callose deposition [47]. Deacetylated pectin is more easily degraded by endogenous and microbial polygalacturonases (PGs), leading to the accumulation of active oligogalacturonide (OG), and the latter functions as damage associated molecular patterns (DAMPs) in plant immunity system for constitutive and pathogen-induced resistance against pathogens [48].

A benzyl alcohol O-benzoyltransferase gene was up-regulated in tobacco leaves during a HR response to a pathogen infection [49]. This enzyme is probably involved in the formation of volatile ester benzylbenzoate, and at least some of these volatile esters may serve as antimicrobial or antifungal agents to prevent further spread of disease [50]. The biotrophic fungal pathogen *Cladosporium fulvum* contains a virulence factor (Avr2) that inhibits several host proteases (including Cys proteases, thiol proteases Aleurain, and Aleurain-like) required for plant basal defense [51].

NSP1 is a member of the GRAS transcription factor family. GRAS transcription factors are proposed to have high functional diversity and may act as integrators of multiple growth regulatory and environmental signals in non-legume plants. GRAS transcripts accumulated in tomato during incompatible interactions and silencing one of them impaired host resistance to bacterial speck disease [52]. The presence of NBS-LRR type R genes is possibly connected to repression of GRAS transcriptional regulators during interactions of potato (*Solanum tuberosum*) with *Phytophthora infestans* [53]. All of these results suggest that the *Cr4*-linked candidate genes may have putative roles in the plant immune system at different levels. However, none of these candidates may encode for an R protein directly involved in recognition of the *C. ribicola* avirulence factor (termed as *avcr4*), and the physiological roles of these genes in *Cr4*-mediated resistance still has to be investigated.

### Potential application of *Cr4*-LG maps in MGR breeding

SNP markers of the *Cr4*-LG provide targeted genomic DNA sequences to develop MAS tools for *P. flexilis* breeding and conservation programs. The match rate of SNP genotypes with the phenotypic traits is an important factor for considering application of SNP markers in practical selection of desired plant traits and prediction of related phenotypic development [29], which depends on genetic distances between diagnostic markers and targeted genes that control the phenotypic trait. Ideal DNA markers should be valid at the species level across all geographic regions the plant species is distributed. A perfect match between genotypes of SNPs and the *Cr4*-mediated resistant phenotype was not detected consistently in all four mapped seedling families in the present study. The numbers of populations and SNPs were still limited for discovery of NBS-LRR or RLK genes with tight linkage to *Cr4*. Due to complex genomes, long life cycles, and difficulty in phenotyping, similar limitations were encountered in genetic mapping of other forest species [14–16, 25, 30, 39]. Therefore, more research is needed to achieve complete accuracy of phenotype prediction within a molecular breeding program of a forest species. Functional SNPs within the *Cr4* gene itself would be an ideal genomic tool for MGR selecting in *P. flexilis*, but positional cloning and functional determination of a conifer gene still requires considerable research effort. Nonetheless, following molecular characterization of R genes, R allele-specific sequences have been used to create marker-assisted tools for plant breeders to select resistance genotypes [54].

The present work identified SNP marker snp290363Y closest to *Cr4* in the seed family GE-213 (with distance of ~ 0.3 cM), but it did not segregate in other three mapped seedling families. Searching additional SNPs from the gene contig_290363, or other *Pinus* conserved genes with physical linkage to this gene in the *P. taeda* genome sequence, may lead to identification of DNA markers for *Cr4* selection with application in a wider range of *P. flexilis* populations.

Markers snp296815Y and snp204077R1 segregated in all four mapped seedling families and placed at loci to *Cr4* at genetic distance of 1.12 and 3.21 cM on the consensus *Cr4*-LG. The same allele at these two SNP loci was linked in coupling to the resistant allele at the *Cr4* locus in all four mapped seedling families, suggesting there may be a potential to use the SNP markers in selection of resistant progeny in related seedling families. It waits further confirmation if these two SNP markers are valid or not in more geographic regions. Using genome-wide genetic markers, genomic selection has the potential to improve *P. taeda* breeding by shortening the current breeding cycle from 12 to 20 years to less than 7 years [55]. Currently it takes 1 ~ 2 years for MGR screening in five-needle pines by artificial rust inoculation in greenhouses. Application of DNA markers will decrease selection time to one or two months as MGR prediction can be performed at the seed germination stage using a segment of cotyledons before limber pine seedlings are planted.

For many of the high elevation five-needle pines, including *P. flexilis*, little breeding work is done. There will be selection, identification of resistant parents (MGR and partial resistance); then management strategies will be developed accordingly [56]. In many cases seed is collected from wild populations, or from resistant parents to use for restoration or reforestation. Therefore, a technique like MAS which requires only needle collections, rather than cone collections followed by progeny tests, can greatly accelerate the identification of resistant seed trees across the landscape. For *P. flexilis*, genomic-based tools can assess the *Cr4* status of younger non-reproductive trees in the field currently untestable with progeny tests, enabling timely assessments of the presence and frequency of *Cr4* in populations that have sustained high mortality of mature trees as a result of disturbance such as the recent mountain pine beetle (*Dendroctonus ponderosae*) epidemic. Estimates of the frequency of *Cr4* in stands and knowing how many MGRs there are in the species would help guide management actions [56]. In addition, the ability to confirm the *Cr4* status of a formerly disease-free tree with recent WPBR symptoms allows for rapid detection of the evolution of virulence (*vcr4*) in *C. ribicola*.

The SNP marker snp204077R1 results in an amino acid change (Lys/His) of galactinol synthase 1, suggesting it may be functional marker affecting enzymatic activity. *Arabidopsis* galactinol synthase is stress-inducible and plays a key role in the accumulation of galactinol and raffinose as osmoprotectants in drought-stress tolerance of plants [57]. Vogan and Schoettle [58] found that *Cr4* seedling families constitutively exhibited greater cold hardiness and lower stomatal conductance than susceptible seedling families during moderate drought. These results suggest that *Cr4* and drought-tolerance genes may be co-inherited and there is a potential to select *P. flexilis* genotypes for better fitness to both biotic and abiotic stresses under a warming climate.

In addition to host markers for marker-assisted selection, pathogen effectors are now applied as molecular markers that accelerate and improve plant breeding of genetic resistance to various pathogens/pests [28]. As the *C. ribicola* reference transcriptome has been assembled and putative pathogenic effectors were identified [59], the *avcr* candidates can be used as pathogenic baits for biochemical screening of their corresponding R products in the five-needle pine. Molecular elucidation of the gene-for-gene model during five-needle pine-blister rust interactions is another strategy that could lead to the identification of the R genes in the WPBR pathosystems.

## Conclusions

The present study represents the first research on genetic mapping of the *P. flexilis* major gene (*Cr4*) for resistance against *C. ribicola* by genotyping SNPs of candidate genes discovered by RNA-seq analysis. The first SNP dataset with more than 100,000 novel SNPs were uncovered in the *P. flexilis* exomes from three seedling families. A large number of non synonymous SNPs for amino acid changes in the encoded proteins provide novel insight into the mechanisms underlying the genetic variability of pest/pathogen resistance and other adaptive traits.

## Methods

### Plant materials and fungus inoculation

For *Cr4* genetic mapping, seeds of four maternal trees GE-213, GE-214, LJ-112, and PHA-106, which have shown segregation of the *Cr4*-controlled canker-free trait in previous tests [12], were stratified in April and sown in May 2014 at Dorena Genetic Resource Center (Dorena-GRC). Megagametophyte samples were collected from each seedling during the seed germination stage, and subject to genomic DNA extraction for *Cr4* genetic mapping based on haploid segregation analysis. Seedlings were inoculated with *C. ribicola* September 8-15, 2014 at Dorena-GRC following a previously reported protocol [12]. After inoculation, seedlings were moved to the greenhouse. WPBR disease symptoms were assessed for each inoculated seedling four times on January 12, February 18, April 22, and November 15, 2015.

For RNA-seq analysis, two susceptible seedling families (MRO-3501 and UT-3359A) and one MGR seedling family (NR-3647) were sown in April, 2010. The MGR seedling family was inoculated in Sept. 2010 and susceptible seedling families were not inoculated. Foliage from at least ten canker-free MGR-survivors or ten non-inoculated susceptible seedlings per seedling family was sampled using liquid nitrogen on July 21–22, 2014 at Dorena-GRC, and kept at -80 °C until RNA extraction.

### Transcriptome assembly by RNA-Seq analysis

Needle samples of the three seedling families were used for total RNA extraction following a manual protocol as described previously [31]. After removal of genomic DNA by DNase (RNase-free) treatment for 30 min at 37 °C, total RNA was cleaned using an RNeasy Plant Mini Kit (Qiagen, Toronto, ON) and quantified using a NanoDrop Spectrophotometer ND-1000 (NanoDrop Technologies, Inc.). RNA integrity was assessed using a 2100 Bioanalyzer (Agilent Technologies). Total RNAs were pooled from 10 seedlings per seedling family. Messenger RNA was separated from 250 ng of total RNAs and cDNA libraries were constructed using a TruSeq stranded mRNA Sample Preparation Kit as per the manufacturer’s recommendations (Illumina). Libraries were quantified using the Quant-iT™ PicoGreen® dsDNA Assay Kit (Life Technologies) and the Kapa Illumina GA with Revised Primers-SYBR Fast Universal kit (D-Mark). Average size of cDNA fragment was estimated using a 2100 Bioanalyzer (Agilent Technologies). Each library was indexed by a sample-specific bar-coding tag and cDNA libraries were pooled in equal ratios for one lane run with 100-bp paired-ends (PE) using Illumina HiSeq2000 at the Génome Québec Innovation Centre, McGill University (Quebec, Canada).

Raw reads were first trimmed using Trimmomatic with default settings at ILLUMINACLIP:TruSeq3-PE.fa:2:30:10 LEADING:3 TRAILING:3 SLIDINGWINDOW:4:15 MINLEN:36 [60]. Trimmed reads from the three seedling families were used to generate a preliminary assembly by *de novo* assembly using Trinity (version: trinityrnaseq_r2013-02–25) with default k-mer length of 25 [61].

*De novo* assembled transcripts were annotated using the BLAST2GO program [62]. The *P. taeda* proteome (high quality protein sequences-9,024 and low quality protein sequences-75,528) derived from genome v1.01 [27] was used as a local database in BLAST programs. *Pinus* highly conserved genes were identified in BLASTx at homology E values ≤ 10e-100. *P. monticola* resistance gene analogs of the NBS-LRR and RLK families and defense responsive genes to *C. ribicola* infection [31] were used in BLASTp programs to search for corresponding homologs in the *P. flexilis* transcriptome.

### Global gene expression analysis

Transcriptome profiles were compared among three seedling families. Trimmed reads from each seedling family were mapped to the *de novo* assembled transcriptome and only paired reads (fragments) were counted in read mapping with a minimum length fraction of 0.9 and a minimum similarity fraction of 0.9. Expression values were calculated as FPKM (Fragments Per Kilobase of exon per Million fragments mapped) using CLC Genomics Workbench 5.5 (CLC bio, QIAgen, Aarhus, Denmark). A resistant seedling family was used as reference group in comparisons. A Z-test was used for statistical analysis with FDR correction to identify differentially expressed genes (DEGs) with fold change > ±2.

### SNP discovery and validation by high-throughput genotyping

*In silico* SNP detection was performed in each seedling family by mapping trimmed RNA-seq reads back to the *de novo*-assembled transcriptome using CLC Genomics Workbench (v5.5) with the following parameters: a mismatch cost of 1, indel cost of 3, length fraction of 0.95, and similarity fraction of 0.95. SNPs were detected using quality-based variation detection at the following parameters: neighborhood radius = 5, maximum gap and mismatch count = 1, minimum neighborhood quality = 20, minimum central quality = 20, ignore non-specific matches = yes, ignore broken pairs = no, minimum coverage = 10, minimum variant frequency (%) = 35, maximum expected alleles = 2, require presence in both forward and reverse reads = yes. The number of shared and unique SNPs was calculated based on pairwise comparisons among three seedling families.

A subset of SNPs were selected from the *in silico* data set generated above for high-throughput genotyping based on a few criteria related to putative gene functions, transcript expression, and SNP types as outlined in a previous report with a little modification [20]. In general, candidate genes with putative functions in disease resistance (the NBS-LRR and RLK gene families) and plant defense as related to WPBR infection, identified by BLAST searches as above, were the targets for a selection of SNPs. Among candidate genes, those *Pinus* highly conserved genes were included with a high priority for a comparative genetic mapping between different *Pinus* species. Of SNPs within candidate genes, we focused on those non-synonymous SNPs, which resulting in dramatic changes of amino acids (for example, changes between neutral amino acids and acidic or basic ones) for a potential to include putative functional SNPs in the genotyping arrays.

Megagametophyte tissues of individual seedlings were homogenized in liquid nitrogen using a FastPre-24 instrument (MP Biomedicals, Santa Ana, CA, USA). Genomic DNA was extracted using a DNeasy Plant Mini kit (Qiagen). SNP genotyping was performed using Sequenom iPLEX MassARRAY (Sequenom, Diego, CA, USA) at the Génome Québec Innovation Centre, McGill University as described previously [20]. A total of 324 *in silico* SNPs were selected for array design and screened for marker segregation analysis using 96 megagametophyte samples from three MGR seedling families, each seedling family with 16 susceptible and 16 resistant samples. Genes and their SNPs with the expected Mendelian segregation ratio (1:1) and potential association with the susceptible phenotype were selected and genotyped in all megagametophyte samples of four seedling families using Sequenom iPLEX SNP genotyping technology.

### Genetic map construction

Haploid segregation analysis was performed to map SNP markers for construction of *Cr4*-LG as described previously [15]. Each SNP marker was tested for Mendelian segregation in each mapping population by *X*^2^ (α = 0.05). Markers showing significant (*P* < 0.05) segregation distortion were initially eliminated from the map construction and were then added later as accessory markers. The polymorphic SNP markers were mapped to *Cr4* using JoinMap version 3.0 software [63]. A LOD threshold of 6 and a distance threshold of 30 cM were used to define a LG. The Kosambi mapping function was used to calculate genetic distances. The consensus map was constructed directly from the marker names and genetic distances in the input maps using MergeMap [64]. The latest *P. taeda* consensus maps [30] were used as references to anchor *Cr4* in *Pinus* LGs. The Pearson correlation coefficient was used to measure the strength of the genetic map relationship between two *Pinus* species. The sequence and nucleotide variation has been submitted to GenBank dbSTS and dbSNP databases.

## Additional files

Additional file 1: Table S1. Statistics of *Pinus flexilis* needle transcriptome *de novo* assembled using TRINITY. **Table S2.** BLAST analysis of *Pinus flexilis* needle transcriptome *de novo* assembled by RNA-seq. **Table S3.** *Pinus flexilis* SNP sequences for design of Sequenom iPLEX MassARRAY. **Table S4.** *Pinus flexilis Cr4*-linked genes showing homologies with *P. taeda* genes mapped on the *Pinus* consensus linkage group 8 (LG8). (XLSX 22 kb) Additional file 2: Figure S1. Comparisons of orders of *Pinus* conserved genes between *P. flexilis Cr4* linkage group (LG) and LG8 of *P. taeda* consensus maps. *P. taeda* LG-8 was revised based on mapping data reported by Westbrook et al. [30]. (PPTX 328 kb)
