# Supplementary figures and images for: Genetic mapping of Pinus flexilis major gene (Cr4) for resistance to white pine blister rust using transcriptome-based SNP genotyping
Source: BMC Genomics. 2016 Sep 23;17:753. doi: 10.1186/s12864-016-3079-2 (PMC5034428; doi:10.1186/s12864-016-3079-2)

## Slide 1
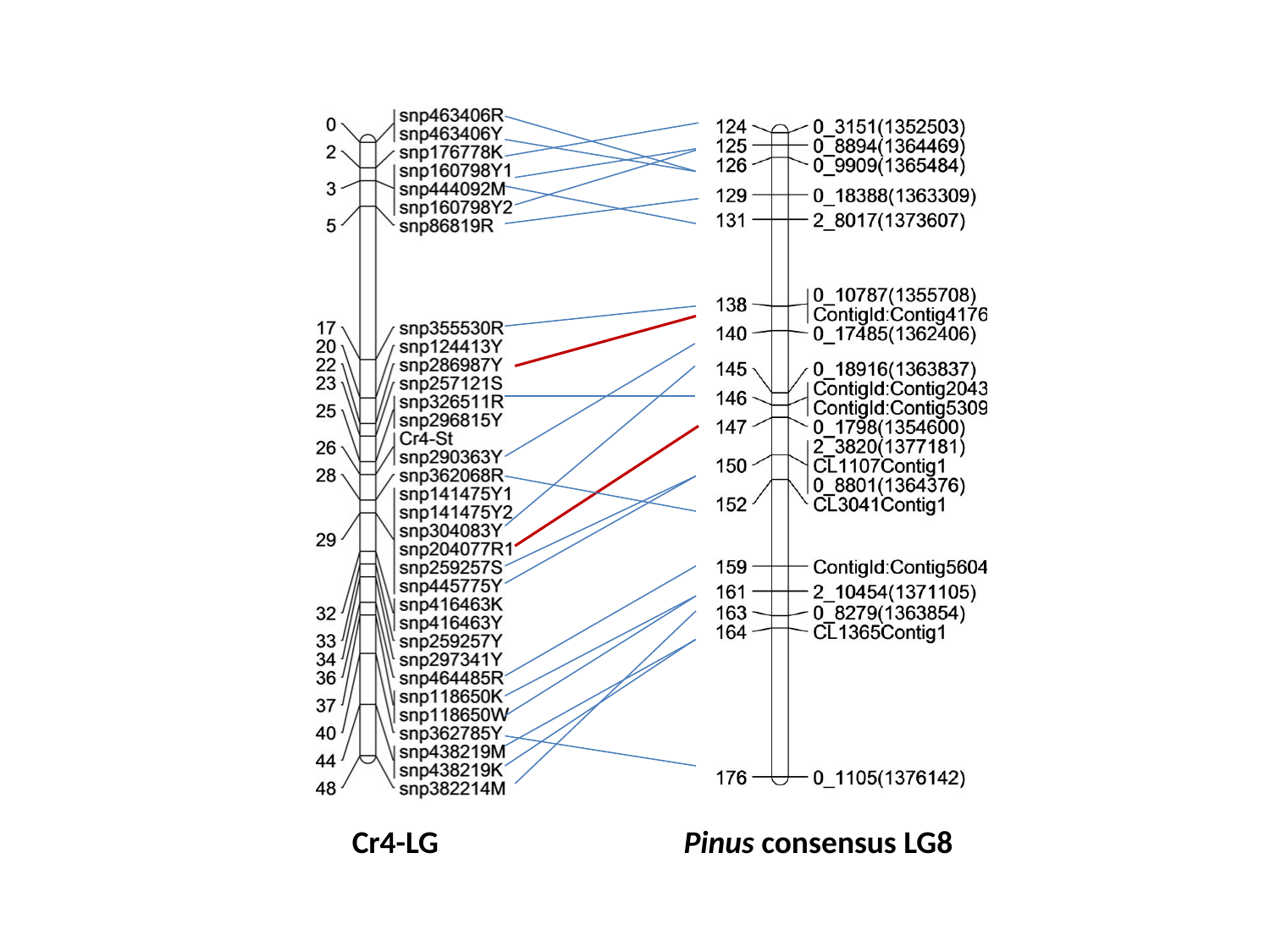

Cr4-LG Pinus consensus LG8

Supplement: Additional file 2: Figure S1. — Comparisons of orders of Pinus conserved genes between P. flexilis Cr4 linkage group (LG) and LG8 of P. taeda consensus maps. P. taeda LG-8 was revised based on mapping data reported by Westbrook et al. [30]. (PPTX 328 kb) [file 12864_2016_3079_MOESM2_ESM.pptx]
